# Transcriptome profiling reveals tissue-wide gene expression in chili pepper (*Capsicum annuum* L.) under infection by *Phytophthora capsici*

**DOI:** 10.3389/fpls.2026.1745952

**Published:** 2026-02-26

**Authors:** Dennis Nicuh Lozada, Patricia Cabrales-Arellano, Jerlie Mhay Matres, Jorge Gil C. Angeles, Victor Velazquez-Martinez, Navdeep Kaur, Efren Delgado, Soum Sanogo

**Affiliations:** 1 Department of Plant and Environmental Sciences, New Mexico State University, Las Cruces, NM, United States; 2 Department of Biology, Eastern New Mexico University, Portales, NM, United States; 3 Philippine Genome Center - Program for Agriculture, Livestock, Fisheries and Forestry, University of the Philippines Los Baños, Laguna, Los Baños, Philippines; 4 Facultad de Ingenieria Mecanica Electrica, Universidad Veracruzana, Poza Rica, Veracruz, Mexico; 5 Department of Horticultural Sciences, Texas A&M University, College Station, TX, United States; 6 Department of Family and Consumer Sciences, New Mexico State University, Las Cruces, NM, United States; 7 Department of Entomology, Plant Pathology and Weed Science, New Mexico State University, Las Cruces, NM, United States

**Keywords:** candidate genes, disease resistance, functional genomics, gene expression, phytophthora root rot, RNA-sequencing

## Abstract

Phytophthora blight, manifested by root, stem, and fruit rot, caused by the oomycete *Phytophthora capsici*, is an important disease affecting Chili pepper production globally. RNA sequencing (RNA-seq) was performed to identify differentially expressed genes (DEGs) and shared genetic resistance mechanisms across different tissues upon infection by the pathogen. RNA-seq revealed the dynamic transcriptome of leaf, stem, and root tissues from resistant (R; CM-334) and susceptible (S; Early Jalapeño) varieties under different times of infection by *P. capsici*. There were 149,531 differentially expressed genes (DEGs) from 39 different R vs. S, time vs. time, and tissue vs. tissue comparisons. A total of 75,520 DEGs (51%) showed higher expression, whereas 74,011 (49%) demonstrated lower expression across all tissues and times of post-inoculation. The total number of DEGs with higher expression for the different tissue samples decreased across times of post-inoculation, where the 72h post-inoculation showed the least number of genes. The roots generally showed a higher number of DEGs compared to the stems and the leaves. Network analyses of DEGs demonstrated that genes with functions related to defense response to fungal infection were also involved with carbohydrate metabolism and ADP binding. Genes related to immune response to fungal infection and amino acid metabolism (e.g., homoserine kinase activity) showed higher gene expression across all times of infection and tissue samples. Chili pepper transcriptome under *P. capsici* infection provides evidence of shared gene expression across multiple tissues which can be leveraged for breeding and selection for broad-spectrum resistance in current *Capsicum* germplasm.

## 1 Introduction

For more than one hundred years, Phytophthora blight, caused by the oomycete *Phytophthora capsici*, has been one of the major diseases affecting Chili pepper (*Capsicum* spp.) production in many growing areas of the world (Quesada-Ocampo et al., 2023; Sanogo et al., 2023). First described by L. H. Leonian in the state of New Mexico, USA (Leonian, 1922), this disease continues to threaten chili pepper production resulting in losses of up to 100% in infected fields. *P. capsici* has been ranked as one of the top five plant-pathogenic oomycetes with scientific and economic importance, alongside *P. infestans, Hyalopernospora arabidopsis, P. ramorum*, and *P. sojae* (Kamoun et al., 2015). *P. capsici* has a broad and expanding host range including plant species in the Cucurbitaceae, Fabaceae, Malvaceae, and Solanaceae families (Erwin and Ribeiro, 1996; Lamour et al., 2012). Breeding for disease resistance remains the most economical approach to mitigate the effects of *P. capsici* in pepper and other host plants. Modern omics tools and integrated management strategies have the potential to render a better understanding of the genetic architecture of the disease enabling more informed and robust breeding and selection decisions for improving disease resistance (Lozada et al., 2022; Sanogo et al., 2023).

As a pathogenic oomycete, *P. capsici* has been known to infect various parts of the chili pepper plant, including the root and stem causing root rot and stem blight (Sy et al., 2005; Kang et al., 2022b). These disease syndromes are influenced by the host species, environmental conditions, and points of infection (Barchenger et al., 2018a). A landrace from Mexico, ‘Criollo de Morellos-334’ (CM-334), has broad-spectrum resistance to *P. capsici*, and has been regarded as a universally resistant host (Reyes-Tena et al., 2019). CM-334, nonetheless, has little to no market and/or agronomic value. Other sources of resistance including pasilla, piquin, cola de rata, and manzano types have been previously identified (Retes-Manjarrez et al., 2020). More recently, Tipo Ancho, Chilhuacle Orange, and Tipo Pasilla have been identified to possess broad-spectrum resistance to three different isolates of *P. capsici* (Kaur et al., 2024), providing other resistant sources to examine the *Capsicum*-*P. capsici* pathosystem using various genomewide approaches.

Functional genomics is the development and application of genome-wide experimental approaches to evaluate the function and expression of genes (Hieter and Boguski, 1997). One of its goals is to bridge the gap between genomic sequences (as anchor points) and function to render novel insights into the characteristics of biological systems (Hieter and Boguski, 1997; Werner, 2010). As an approach, RNA-seq has become a standard method for studying gene expression, specifically for evaluating transcript abundance and diversity (Griffith et al., 2015). Previous studies have demonstrated the potential of using RNA-seq approaches to explore the genes and gene systems associated with response against plant infection by *P. capsici*. Different transcription factor and protein families including protein kinases were observed in two contrasting chili pepper landraces (i.e., ‘GojamMecha_9086’ (resistant) and ‘Dabat_80045’ (susceptible)) exposed to *P. capsici* (Rabuma et al., 2022). In another study, candidate genes related to the modification of cell wall, phytoalexins, and phytohormones were identified to be associated with responses to *P. capsici* in the pepper line PI 201234 (Wang et al., 2015). The involvement of the phenylpropanoid biosynthesis pathway has been previously implicated in resistance against *P. capsici* in whole roots of chili pepper (Li et al., 2020) and black pepper (*Piper* spp.) (Hao et al., 2016). The higher expression of enzymes, including proteases (subtilisin-like protease and xylem cysteine proteinase 1), and various pathways such as Ca^2+^- and salicylic acid-mediated signaling, and flavonoid biosynthesis pathways were further observed in response to infection by *P. capsici* (Lei et al., 2023). A total of 14 putative effectors were also previously observed to be differentially expressed between various *Capsicum-P. capsici* interactions, where 12 genes were classified as RxLR (Arginine-any amino acid-Leucine-Arginine) and two as CRN (Crinkling and Necrosis) effectors (Maillot et al., 2022).

In the current study, we have identified differentially expressed genes in various tissues (roots, stems, and leaves) of resistant and susceptible pepper varieties under infection by *P. capsici* using an Illumina-based RNA-seq approach. The differential expression of genes and gene systems across different plant tissues contribute to the overall genetic resistance and/or susceptibility to infection of *Capsicum* by *P. capsici.* Results from gene expression profiling could be utilized for omics-assisted breeding for disease resistance in current chili pepper germplasm.

## 2 Materials and methods

### 2.1 Plant material and inoculation with *P. capsici*

Two varieties, namely, CM-334 (resistant) and Early Jalapeño (susceptible) (*C. annuum* L.) were used in the current study. These varieties were used for transcriptome analyses as they were previously used as parents to develop the New Mexico recombinant inbred lines (NMRIL) population (Sy et al., 2005). The NMRIL has been extensively used to examine the genetics of *P. capsici* resistance in *C. annuum* L. (Monroy-Barbosa and Bosland, 2008; Sy et al., 2008; Jiang et al., 2015; Barchenger et al., 2018b; Lozada et al., 2021a). Seeds were planted in three replications and maintained under standard greenhouse conditions for growing chili pepper at the Fabian Garcia Science Center Greenhouse, New Mexico State University, Las Cruces, NM (Sharma et al., 2017) in March 2022. The greenhouse is comprised of aluminum frames, double layer of polycarbonate sheets for insulation, evaporative cooler, and heaters. The temperature inside the greenhouse is controlled by an automatic control system, with average temperatures of 27.5°C (daytime; mean 12.5 hours) and 21.6°C (nighttime; mean 11.5 hours). At the 4–8 leaf stage, the soil was inoculated with ~10,000 zoospores of 6347, a virulent isolate of *P. capsici* collected from a commercial farm in New Mexico (Jiang et al., 2015), following the methods previously described (Kaur et al., 2024; Lozada et al., 2021a). At times 0-, 24- and 72- hours post infection (hpi), leaf, stem, and root tissues were sampled and stored in DNA/RNA shield buffer for RNA extraction (Zymo Research, Irvine, CA, USA) in April 2022. In total, there were 18 variety-tissue-timepoint combinations [two varieties (resistant vs. susceptible) x three tissues (leaves, roots, and stems) x three timepoints (0, 24, and 72)] in three biological replicates (total *n* = 54 samples).

### 2.2 RNA isolation, library construction, and sequencing

Total RNA was extracted from leaf, roots, and stems of CM-334 and Early Jalapeño at different time points using the Trizol reagent (Invitrogen) following the manufacturer’s instructions. DNA contamination was removed using RNA clean and concentrator (Zymo Research, CA, USA). RNA was quantified using Qubit^®^ RNA Assay Kit (Life Technologies, MA, USA). Total RNA (500–1000 ng) was used for library preparation using poly-A selection method of KAPA mRNA HyperPrep Kits (Roche, CA, USA) following the manufacturer’s protocol. The final concentration of all cDNA libraries was measured using the Qubit^®^ dsDNA HS Assay Kit (Life Technologies). Agilent 2100 Bioanalyzer (Agilent Technologies, CA, USA) was used to determine the average size of the libraries. Paired-end (150-bp) sequencing was performed for all libraries pooled in equimolar ratios of 0.6nM using the Illumina NovaSeq 6000^®^ platform (Illumina, CA, USA). The read files for each sample were analyzed using Cutadapt v4.4 (Martin, 2011) to identify and remove Illumina sequencing adapters. Trimmomatic v0.40 (Bolger et al., 2014) was used to eliminate poor quality reads, or low quality 5’ or 3’ bases or fragments implementing a sliding window approach, where a window of 10 bp was moved from the 5’ to the 3’ end of the read one base at a time. Reads or bases with the following qualities: (a) reads with average quality score < 20; (b) reads < 50 bp in length; and (c) bases with quality scores ≤ 3 at the 5’ or 3’ bases were excluded. Applying these criteria, two samples (viz., S_leaf_24_2 and S_root_72_3) were excluded, resulting in *n* = 52 biological samples for transcriptome analyses. **Supplementary Table 1** summarizes the software tools and packages used for the analyses of RNA-seq data.

### 2.3 Quality control and quantification of transcript number

Following the quality control filtering steps, the number of reads per transcript was quantified using Kallisto v0.46.1 (Bray et al., 2016), which implements a pseudoalignment approach to rapidly determine the compatibility of reads with transcripts. A list of *k*-mers (sequence fragments of length *k*) from the transcriptome were generated using Kallisto. Pseudoalignments were created and quantified through Kallisto by comparing the *k*-mers of each read to the de Bruijn graph model of the transcripts. The *C. annuum* L. reference genome UCD10Xv1.1 (RefSeq GCF_002878395.1; GenBank GCA_002878395.3 Annotation release 101; Hulse-Kemp et al., 2018) was used for the estimation of the number of transcripts. The reads per transcript counts reported by Kallisto were then used to generate reads per gene counts with the ‘tximport’ v1.28 package (Soneson et al., 2016) in R.

### 2.4 Cluster analyses

Principal components analysis (PCA) and Principal coordinates analysis (PCoA) were implemented to visualize the level of similarity of the samples’ transcriptional profiles (i.e., the number of reads assigned to different genes in different samples). Prior to implementing the PCoA and PCA, the reads per gene counts for each sample were normalized using a variance-stabilizing transformation (VST) approach in DeSeq2 v.3.22, which normalizes the data in relation to the library size and removes the dependence of the variance on the mean, where genes with low mean counts show higher variance than genes with high mean counts (Love et al., 2014). The transformation measures and removes the experiment-wide trend of variance over mean. The PCA was performed using a singular value decomposition of the normalized counts per gene matrix (Venables and Ripley, 2013). The normalized counts were used to calculate the Euclidean distances between each pair of samples and the distance matrix was used as input for PCoA (Gower, 1966). The ellipses for groups with more than 3 samples in the PCoA plots show the 95% confidence level for a multivariate *t*-distribution (Fox and Weisberg, 2011; Friendly et al., 2013).

### 2.5 Identification and functional annotation of differentially expressed genes

Genes that demonstrated significant expression changes across different groups of samples (pairwise comparisons/contrasts) were identified using DESeq2, where a Wald test with Benjamini-Hochberg correction for sampling was performed (Love et al., 2014). Genes with adjusted *P* ≤ 0.05 and log_2_ of fold change (log_2_FC) ≥ 1 and ≤ -1 were considered to have higher expression and lower expression, respectively. Functional annotation was performed using different information including (a) the protein product obtained from the general feature format (GFF) annotations for the corresponding reference genome and transcriptome in the NCBI RefSeq database; (b) KEGG pathways (Kanehisa and Goto, 2000); and (c) Gene ontology (GO) terms retrieved from the UniProt database using the ‘AnnotationHub’ package (Morgan et al., 2019) in R. Heatmaps showing expression changes (fold changes, FC) for the DEGs based on their corresponding log_2_FC values were obtained using the ‘pheatmap’ package (Kolde and Kolde, 2015) in R. Enrichment of biological functions among the DEGs were performed using a hypergeometric test and *P*-values were adjusted for multiple testing using the Benjamini-Hochberg correction.

## 3 Results

### 3.1 RNA quality and summary of RNA sequencing

The total RNA concentration for the samples (*n* = 54) ranged between 33.8 ng/ul and 5,747 ng/ul, with a mean value of 486.30 ng/ul. The average RNA integrity number (RIN) value for a random subset of the samples (*n* = 21) was 9.03, with a range between 7.1 and 9.8. A total of 592,493,398 reads from *n* = 54 samples were subjected to transcriptome profiling (**Table 1**). Out of this number, 447,084,240 (75.5%) were pseudoaligned using Kallisto. Non-duplicate (unique) reads comprised 55.2% of the total number of reads (327,117,203). The average number of reads for the 54 samples was 10,972,100 (**Supplementary Table 2**). Considering *n* = 52 samples, there were 562,528,911 total reads, of which 437,616,790 (77.8%) were pseudoaligned. The number of unique reads was 319,695,213 (56.8%), with an average number of 10,817,864 reads, for *n* = 52 samples.

**Table 1:** Summary of RNA sequencing results.

Total number of samples	Total number of reads	Average number of reads	Total number of pseudoaligned reads	Pseudoaligned reads (%)	Number of unique (non-duplicate) reads	Unique reads (%)
54	592,493,398	10,972,100	447,084,240	75.5	327,117,203	55.2
52	562,528,911	10,817,864	437,616,790	77.8	319,695,213	56.8

### 3.2 Differential expression of genes

There were 149,531 differentially expressed genes (DEGs) (*P* ≤ 0.05 and log_2_ of fold change (log_2_FC) ≥ 1 and ≤ -1 for high gene expression and low gene expression, respectively) representing 39 different contrasts/comparisons from 52 biological samples subjected to RNA-seq analyses. Different versions of the same gene (i.e., isoforms) were counted separately. The Top 100 differentially expressed genes across the different tissues and hours post-inoculation are listed on **Supplementary Tables 3**-**10**. A total of 75,520 (51%) DEGs showed higher expression, whereas 74,011 (49%) DEGs showed lower expression in the R vs. S contrast. Excluding the transcripts in unplaced (unmapped) scaffolds, there were 134,753 DEGs, with 68,231 (51%) showing higher expression and 66,522 (49%) demonstrating lower expression. Except for the stems, the total number of DEGs with higher expression for the different tissue samples decreased across the different times of post-inoculation, where the 72-h post-inoculation showed the least number of genes. Comparing the resistant (R) vs. susceptible (S) varieties across different tissues, a total of 3,652, 3,037, and 2,069 differentially expressed genes (DEGs) were identified in the roots, stem, and leaves, respectively. In the roots, 1,535 genes showed higher expression, and 2,117 genes were observed to have lower expression; in the stem, 1,446 demonstrated higher expression and 1,591 genes showed lower expression; and in the leaves, 907 genes showed higher expression, and 1,162 genes demonstrated lower expression.

**Figure 1:**
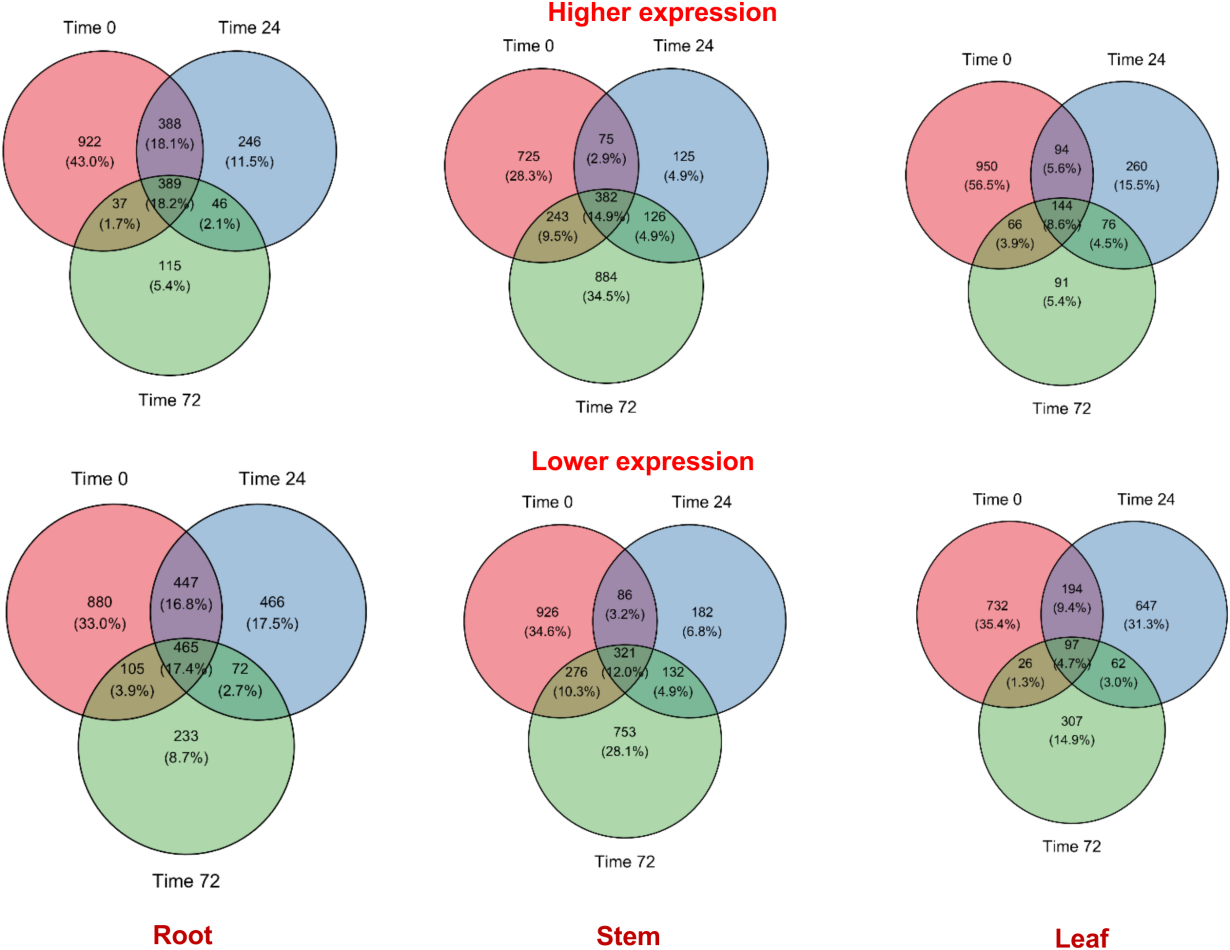
Number of differentially expressed genes across different times (hours, h) post-infection with *P. capsici* (0-, 24-, and 72-h for the resistant (R; CM-334) vs. susceptible (S; Early Jalapeno) contrast for root, stem, and leaf tissue samples. .

Comparing the R vs. S varieties across different times of post-inoculation, a total of 1,736 DEGs showed higher expression in the roots at 0h (**Figure 1**). The number of DEGs with higher expression decreased to 1,069 at 24h, and to 587 at 72h post-inoculation with *P. capsici*. A total of 922, 246, and 115 genes were unique to 0h, 24h, and 72h post-inoculation, respectively, whereas 389 genes were shared in all timepoint comparisons. In comparison to the root tissues, the total number of genes with higher expression was relatively less in the stems and the leaves (2,560 and 1,681 DEGs, respectively). For the stems, the number of distinct genes with higher expression was 725 at 0h, 125 at 24h, and 884 at 72h, whereas in the leaves, there were 950 (0h), 260 (24h), and 91 (72h) genes that showed higher expression. A total of 382 and 144 DEGs with higher expression were shared at all timepoint comparisons for the stem and leaves, respectively.

At different times of infection in the R vs. S contrast, 2,668 genes showed lower gene expression in the roots, whereas 2,676, and 2,065 genes were observed to have lower expression in the stems and leaves, respectively. The number of genes with lower expression also decreased over time of post-inoculation for the root and leaf tissues, showing a similar trend with the observation for the genes with higher expression. In the root, 880 unique genes showed lower expression at 0h post-inoculation (hpi) whereas, 466 unique genes were observed to have lower expression at 24hpi. The number of unique DEGs with lower expression further decreased to 233 at 72hpi. A total of 465 DEGs in the root tissue were common across all timepoints. The number of DEGs with lower expression was higher in the stem than the root (2,676), with 926, 182, and 753 unique genes for the 0h, 24h, and 72h post-inoculation, respectively. A total of 2,065 genes showed lower expression in the leaf tissues, with 97 genes common across all the times of post-inoculation. Overall, there were 732 genes with lower expression unique for 0h; 647 for 24h; and 307 for 72h in the leaf tissue after inoculation with *P. capsici*.

### 3.3 Principal components analysis and multidimensional scaling

**Figure 2:**
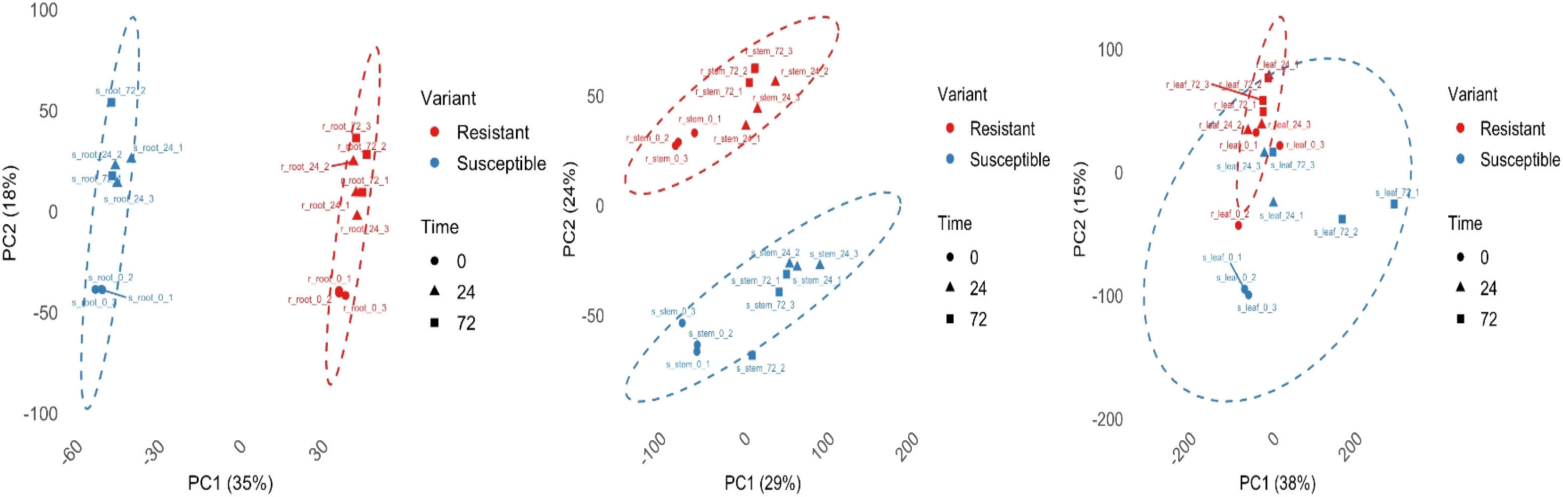
Principal components analysis (PCA) biplots of the transcriptomes of CM-334 (resistant) and Early Jalapeno (susceptible) across various times (*t*) of *P. capsici* infection (0, 24, 72 h) for **(a)** root, **(b)** stem, and **(c)** leaf tissue samples.

Principal components analysis (PCA) was implemented to examine the relationships between the transcriptional profiles of the samples. Samples S_leaf_24_2 and S_root_72_3 (Susceptible_Leaf_ 24hpi_biological replicate 2 and Susceptible_Root_72hpi_biological replicate 3, respectively) and were identified as outliers by visual inspection of the PCA clustering and hence were excluded from further analyses. Clustering based on tissue type was observed when all samples were analyzed using PCA (**Figure 2**). The first and second principal components (PC1 and PC2) were associated with 35% and 18% of variation, respectively, for the root samples. Grouping based on resistance or susceptibility was most apparent in roots and stems, whereas no clear clustering was observed among the leaf samples. The transcriptional profiles for the varieties were more similar prior to inoculation with *P. capsici* (0h), relative to after inoculation at 24hpi and 72hpi. The transcriptome at 0hpi tends to form a tight cluster within the resistant and susceptible varieties across the different tissues, whereas there was no clear grouping that could be observed for RNA-seq profiles at 24hpi and 72hpi.

In addition to the PCA, the level of similarity between the transcriptional profiles between the samples was visualized using principal coordinates analysis (PCoA) (**Supplementary Figure 1**). Clustering based on the tissue source and not based on resistance or susceptibility of the varieties, was apparent when all samples were analyzed altogether using PCoA. When samples were grouped based on resistance or susceptibility, PCoA clustering was more pronounced in the root samples, where the transcripts of the resistant variety grouped together. Conversely, grouping based on resistance/susceptibility was less observable in the stem samples and was not apparent in the leaf samples. More robust clustering between samples at 0hpi was observed, relative to samples at 24hpi and 72hpi when PCoA was performed based on inoculation time.

### 3.4 Differentially expressed genes with higher expression

**Figure 3:**
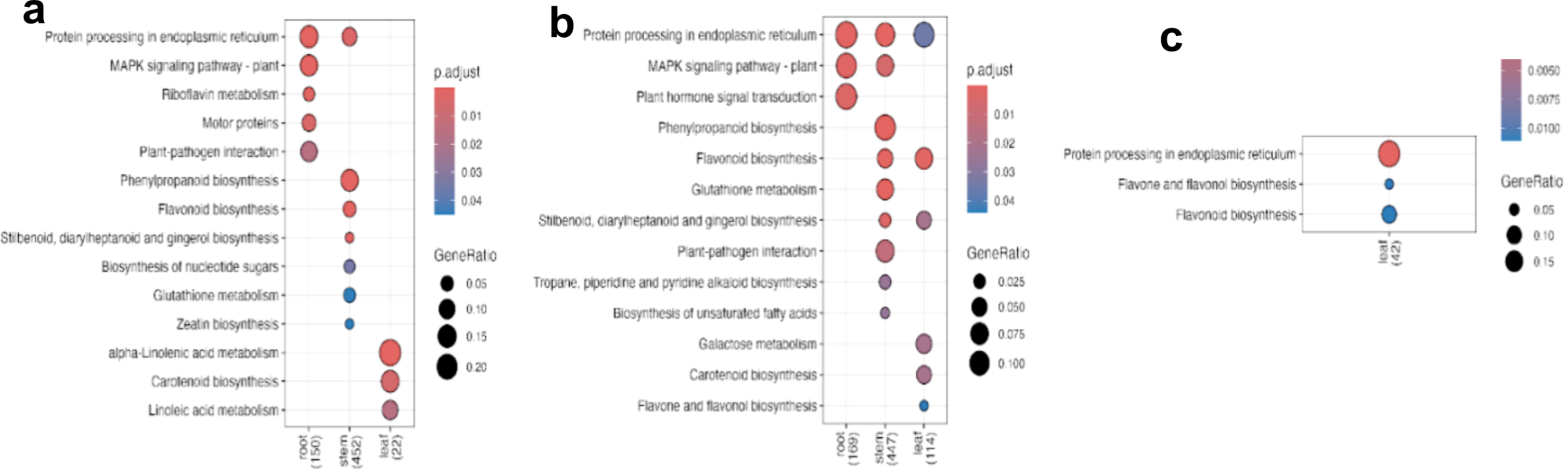
Bubble plots showing the significantly enriched KEGG functions for various time (hours, *h* post-inoculation) contrasts in CM-334 (resistant variety): **(a)** 24h/0h, **(b)** 72h/0h, and **(c)** 72h/24h across all tissue samples.

The differentially expressed genes with higher expression for the R vs. S contrast belong to different chromosomes including chromosomes 1, 2, 5, 7, 11, and 12 (**Table 2**). In the roots, the mean log_2_FC value was highest for Zeatin O-xylosyltransferase with a UDP-glycosyltransferase activity in chromosome 5. This gene was identified on the *Ext-Pc5.1* region for *P. capsici* resistance previously described by Du et al. (2021). Similarly, homoserine kinase also showed higher expression and was mapped within the *Ext-Pc5.1* region. Genes with roles related to glutathione metabolic process, glutathione transferase activity, ATP-dependent DNA damage activity, also demonstrated higher expression. In the resistant variety (CM-334) across time vs. time comparisons, genes with biological functions associated with systemic acquired resistance (e.g., putative lipid-transfer protein *DIR1*), defense response to other organisms (e.g., pathogenesis-related protein *R* major form; pathogenesis-related protein *P2*; putative late blight resistance protein homolog *R1A-3*; disease resistance protein *RGA2*-like), and defense response to fungus (e.g., acidic endochitinase *pcht28*, Early nodulin-75, pathogenesis-related protein *P2*) showed consistent higher expression across multiple tissues (**Table 3****;** **Figure 3**).

**Table 2:** The top 20 differentially expressed genes with higher expression in roots and their functions.

Gene ID	Chr.	Position (start, end; in bp)	Gene	Function(s)	Average log_2_FC
107870473	5	26240506, 26244754	***Zeatin O-xylosyltransferase***	UDP-glycosyltransferase activity	10.91
107848341	11	32830222, 32831544	Glutathione S-transferase U17	Glutathione metabolic process; glutathione transferase activity	10.56
107877977	7	161977837, 162066908	DNA mismatch repair protein *MSH7*	Mismatched DNA binding; ATP binding; mismatch repair; ATP-dependent DNA damage activity	10.54
107870410	5	20998254, 20999886	***Homoserine kinase***	Homoserine kinase activity; ATP binding; threonine metabolic process	10.17
107850467	12	8368711, 8374174	Beta-glucosidase 42	Carbohydrate metabolic process; beta-glucosidase activity	10.08
107877337	7	210507508, 210509477	Protein NODULATION SIGNALING PATHWAY 2	Sequence-specific DNA binding; DNA-binding transcription factor activity; regulation of DNA-templated transcription	9.34
107860513	2	150028093, 150032914	A-kinase anchor protein 7	DNA dealkylation involved in DNA repair; regulation of DNA-templated transcription	9.16
107875488	1	55362182, 55372791	COP9 signalosome complex subunit 2	Protein deneddylation	9.08
107878033	1	96977394, 96986051	Serine/threonine-protein Phosphatase PP2A catalytic subunit	Peptidyl-serine dephosphorylation; mitotic cell cycle; protein serine/threonine phosphatase activity	8.82
107868168	4	23444894, 23457437	Vacuolar protein sorting-associated protein 8 homolog	Protein binding; endosomal vesicle fusion; late endosome; HOPS complex; protein targeting to vacuole	8.59
107842555	9	65999409, 66002761	Putative glutathione peroxidase 5	Response to oxidative stress; glutathione peroxidase activity	8.31
107859557	2	133594838, 133611861	Pectinesterase	Cell wall modification; pectinesterase inhibitor activity; pectinesterase activity	8.04
107872960	6	175798464, 175799168	Protein cornichon homolog 4	Vesicle-mediated transport	7.95
107868113	4	58273595, 58279483	Transcription factor bHLH62	Regulation of DNA-templated transcription; DNA-binding transcription factor activity	7.88
107848161	11	58156110, 58166643	Putative BOI-related E3 ubiquitin-protein ligase 3	Ubiquitin-protein transferase activity	7.58
107840746	9	217422587, 217424606	Putative late blight resistance protein homolog R1B-13	Protein folding; alpha-tubulin binding	7.53
107857159	1	193609050, 193609671	Putative lipid-transfer protein *DIR1*	Fatty acid binding; systemic acquired resistance	7.30
107871108	5	205442143, 205443599	U4/U6 small nuclear ribonucleoprotein Prp31 homolog	U4/U6 x U5 tri-snRNP complex;U4 snRNP; precatalytic spliceosome; mRNA splicing, via spliceosome; spliceosomal tri-snRNP complex assembly	7.04
107863749	3	68261538, 68269405	Glycine cleavage system H protein	Glycine decarboxylation via glycine cleavage system; protein lipoylation; glycine cleavage complex; DNA binding	6.73
124897050	3	29468788, 29477960	Cyanidin 3-O-glucoside 7-O-glucosyltransferase (acyl-glucose)-like	Beta-glucosidase activity; carbohydrate metabolic process	6.71

Differential expressions based on three contrasts for the resistant (R) vs. susceptible (S) contrast (0h/0h; 24h/24h; 72h/72h post-inoculation). Genes in chromosome 5 (italicized and boldfaced) lie in the extended *P. capsici* genomic region (*Ext-Pc5.1*) previously identified by Du et al. (2021).

**Table 3:** Significantly enriched (higher expression; *P* < 0.05) genes and their corresponding biological processes and functions in CM-334 (resistant variety (time vs. time comparison)) across various tissues. Functions in bold and italics are common in multiple tissues.

Biological processes/functions	Gene	Gene ratio	Contrast(s) (time, *t* in hours post-inoculation)	Tissue
***Systemic acquired resistance***	Putative lipid-transfer protein *DIR1*	0.04	72/0	Root
	Putative lipid-transfer protein *DIR1*	0.05	72/0; 24/0	Stem
	Putative lipid-transfer protein Protein *DIR1*	0.05	72/24	Leaves
Plant-pathogen interaction	RPM1-interacting protein 4	0.1	24/0	Root
	Putative calcium-binding protein *CML19*	0.1	24/0	Root
	Probable LRR receptor-like serine/threonine-protein kinase *At3g47570*	0.1	24/0	Root
	LRR receptor-like serine/threonine-protein kinase *FLS2*	0.1	24/0	Root
Response to ethylene	Ethylene receptor 2	0.03	24/0	Root
	REVERSION-TO-ETHYLENE SENSITIVITY1	0.03	24/0	Root
	Protein *EIN4*	0.03	24/0	Root
***Defense response to other organism***	Protein *SRC2*	0.05	72/0; 24/0	Stem
	MLO-like protein 1	0.05	72/0; 24/0	Stem
	Defensin *J1*-2-like	0.05	72/0; 24/0	Stem
	Bon1-association protein 2	0.05	72/0; 24/0	Stem
	Pathogenesis-related protein R major form	0.1	72/24	Leaves
	Pathogenesis-related protein *P2*	0.1	72/24	Leaves
	Putative late blight resistance protein homolog *R1A-3*	0.1	72/24	Leaves
	Disease resistance protein *RGA2*-like	0.1	72/24	Leaves
***Defense response to fungus***	Acidic endochitinase *pcht28*	0.05	72/24	Leaves
	Early nodulin-75	0.05	72/24	Leaves
	Wound-induced protein *WIN2* pathogenesis-related protein *P2*	0.05	72/24	Leaves
	Ethylene-responsive transcription factor 5-like	0.05	72/0; 72/24	Stem
Cell wall biogenesis	Cellulose synthase-like protein *D3*	0.05	24/0	Stem
	Xyloglucan endotransglucosylase/hydrolase protein 15-like	0.05	24/0	Stem
Response to reactive oxygen species	L-ascorbate peroxidase 2 cytosolic	0.05	24/0	Stem
	L-ascorbate peroxidase 3	0.05	24/0	Stem
	L-ascorbate peroxidase 1 cytosolic-like	0.05	24/0	Stem
Phenylpropanoid biosynthesis	Caffeoyl-CoA O-methyltransferase	0.1	24/0; 72/0	Stem
Plant-pathogen interaction MAPK signaling pathway	Respiratory burst oxidase homolog protein B	0.05	72/0	Stem
	Mitogen-activated protein kinase 3	0.05	72/0	Stem
	WRKY transcription factor 22	0.05	72/0	Stem
	Mitogen-activated protein kinase homolog *MMK2*	0.05	72/0	Stem

### 3.5 Network analyses of differentially expressed genes

**Figure 4:**
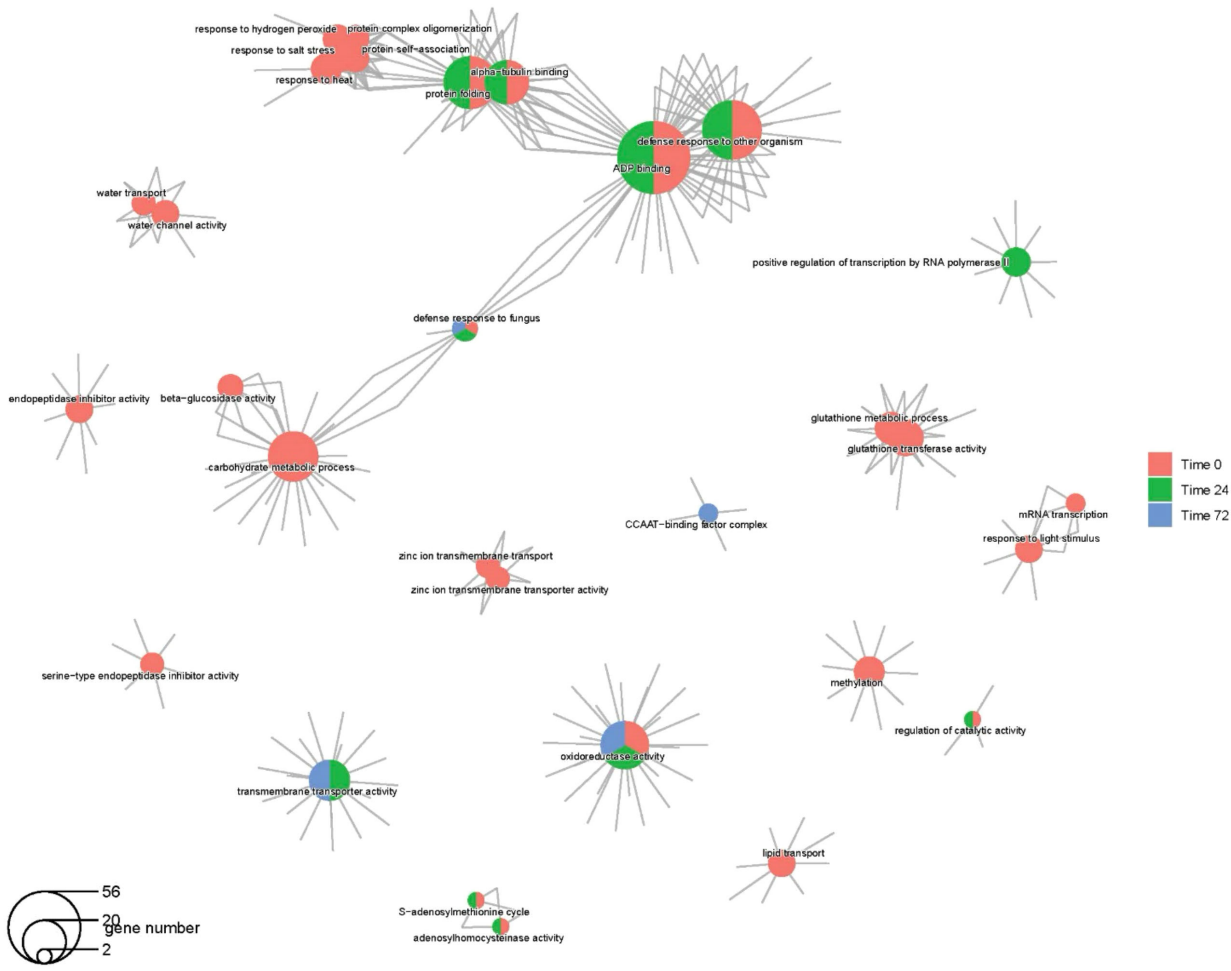
Network of significantly enriched GO functions in resistant (CM-334) vs susceptible (early jalapeño) samples from different tissues. Results from genes with higher expression is shown. The size of a function node is proportional to the number of differentially expressed genes (DEGs) annotated with a given function in all of the comparisons. The DEGs (small nodes) are shown connected to their corresponding functional categories. Function nodes are connected through shared DEGs. The DEG nodes are colored according to their occurrence in different contrasts. A function node is colored by the presence of DEGs annotated with that function in different contrasts, independently of the enrichment level of the function in each contrast.

Genes related to immune response against fungal infection were differentially expressed across all time points. Network analyses of DEGs for the R vs. S contrast demonstrated that genes involved in defense response to fungal infection across all timepoints were also involved with carbohydrate metabolism and ADP binding (**Figure 4**). The DEGs with roles related to defense responses to other organisms were also involved in ADP binding and were differentially expressed in 0hpi and 24hpi with *P. capsici*. Genes with roles related to transmembrane transporter and oxidoreductase activities were differentially expressed in 24- and 72, and 0-, 24-, and 72hpi, respectively. Genes with functions associated with methylation, lipid transport, mRNA transcription, response to light, water transport, and endopeptidase inhibitor activity were differentially expressed only at the 0hpi. Genes involved in positive regulation of transcription by RNA Pol II were differentially expressed exclusively at 24hpi with *P. capsici*.

Genes with functions associated with defense response to other organisms showed higher expression across all tissue samples, and shared roles in binding of ADP and monooxygenase and oxidoreductase activities (**Supplementary Figure 2**). Similarly, genes with roles in protein folding and alpha-tubulin binding were observed to have higher expression in the roots, stems, and leaves, and shared functions with ADP binding. Genes with roles related to protein dimerization, biosynthetic process of aromatic compounds, methyltransferase activity, and response to light showed higher expression exclusively in the leaves, whereas those involved in secondary shoot formation regulation were unique to the stem. Endopeptidase inhibitor activity, transmembrane transport, and transmembrane transporter activity-related genes were observed to have higher expression in the roots whereas genes with functions related to iron binding and DNA-binding transcription activity showed higher expression on both the leaf and stem tissues.

## 4 Discussion

Phytophthora blight caused by *P. capsici* remains one of the most destructive diseases affecting chili pepper production in many areas of the world where the crop is cultivated. Understanding the genomic and transcriptomic basis of plant infection by *P. capsici* will facilitate robust and more informed breeding and selection decisions for improving resistance in current *Capsicum* germplasm (Barchenger et al., 2018a; Lozada et al., 2022). In the present study, gene expression analyses identified DEGs from different tissues and demonstrated the diversity of candidate genes involved in resistance to *P. capsici* in chili pepper. Comparative transcriptomics supported the presence of tissue-wide genetic resistance across different organs and tissue-specific mechanisms of the pepper plant under infection by the pathogen.

Root and stem showed similar transcriptome profiles for each biological replicate, where the resistant (R) and susceptible (S) samples clustered into their respective groups in the PCA biplot. In contrast, the leaf tissue demonstrated less consistent, overlapping clusters between the R and S samples. This could demonstrate relatively delayed and/or weaker immune responses in the leaves, compared to the roots and stems. This differential responses to *P. capsici* infection across the various tissues is consistent with the number of genes with higher expression, where the roots showed the highest number followed by the stem, and the leaf tissues. This could also be a possible consequence of the inoculation method used, where the pathogen was introduced via the soil; therefore, the initial contact occured in the roots.

The decreased number of DEGs in the 72hpi could be due to the temporal nature of the dynamics of the interaction between *P. capsici* and chili pepper. The initial response in the 0-24hpi, for example, could have resulted in higher transcriptional responses in the pepper plant, relative to the 72hpi, where responses have decreased, resulting in a lesser number of DEGs. In contrast to the roots and leaves where the number of genes with differential expression decreased as time of infection progressed, the stems showed an increased number of unique DEGs at 72hpi. The stems among the tissues evaluated for RNA-seq seem to have distinct mechanisms of resistance to *P. capsici* infection compared to the roots and leaves, as more genes with diverse functions showed higher differential expression in the stem (**Table 3**). The genes involved in constitutive or passive defenses (e.g., cell wall formation) were in general differentially expressed in the earlier phase of infection. In the later stage, those genes related to active immunity such as those with roles related to mitogen activated protein kinase signaling pathway, defense response, and systemic acquired resistance were differentially expressed. This indicated a shift from a passive to more active form of immunity as *P. capsici* infection progressed, albeit the number of differentially expressed genes generally decreased.

The majority of the DEGs (>80%) were exclusive to each tissue, indicating varying responses for each different part of the chili pepper plant upon infection by *P. capsici*. It was previously noted that infection of stem, leaf (foliar), and root caused by *P. capsici* demonstrate distinct, multiple disease syndromes, and each tissue will have different modes of action to reduce the effects of the pathogen (Sy et al., 2005). Remarkably, some genes showed higher expression across all tissue samples indicating the presence of shared disease responses among the different tissue types. These include the putative late blight resistance protein homolog *R1B-16*, alpha-crystallin domain-containing protein 22.3-like, putative disease resistance protein *RGA3*, and *CSC1*-like protein *ERD4*, with functions related to defense response to fungus and other organisms, protein kinase activity, and ADP and ATP binding which showed higher expression across all tissues in the R vs. S contrast.

Gene network analyses supported that genes with functions associated with defense responses to other organisms showed higher expression across all tissue samples in the R vs. S comparison. In other plant-pathogen pathosystems, such genes have been identified in the context of both biotic and abiotic stresses. In soybean (*Glycine max*), genes associated with defense, replication of DNA, and iron homeostasis demonstrated differential expression in root, stem, and leaf tissues and were regarded as “hallmarks of resistance” to brown stem rot caused by the fungus *Phialophora gregata* (McCabe et al., 2018). Genes related to phenylpropanoid biosynthesis, phenylalanine ammonia lyase (PAL), and peroxidase enzyme activity observed to have higher expression in the stems, roots, and leaves of black pepper varieties challenged with *P. capsici* (Hao et al., 2016). Recently, signal transduction pathways of plant hormones including abscisic acid (ABA) biosynthesis and signaling related genes (9-cis-epoxycarotenoid dioxygenase (*NCED*), phosphatase 2C (*PP2C*), *SNF1*-related protein kinase 2 (*SnRK2*), and ABA-responsive element binding factors (ABF)) were observed to have higher expression in both leaf and root tissue samples in response to drought stress in river tamarind (*Leucaena leucocephala*) (Zhi et al., 2024).

Chromosome 5 is a known major genomic hotspot for *P. capsici*-resistant QTLs and metaQTLs in chili pepper (Chunthawodtiporn et al., 2019; Kaur et al., 2024; Liu et al., 2014; Lozada et al., 2021a, 2021b; Mallard et al., 2013). An extended disease resistance QTL region, *Ext-Pc5.1*, pinpointed at 8.35 Mb–38.13 Mb on chromosome 5, has been previously described (Du et al., 2021). In the current study, transcriptomic analyses across all tissue samples revealed the higher expression of a gene located within this chromosome 5 hotspot between ~20.99 and 21 Mb with a homoserine kinase (HSK) activity function. The *C. annuum* downy mildew resistance 1 gene (*CaDMR1*), an HSK, and an orthologue of *DMR1* that catalyzes the conversion of homoserine to homoserine-4-phosphate, a step in the aspartic acid-derived threonine biosynthetic pathway in *Arabidopsis* (van Damme et al., 2009) has been previously identified as a candidate gene for resistance to *P. capsici* at ~24.36 Mb in chromosome 5 (Rehrig et al., 2014). Due to the colocalization of the HSK gene identified in the present study with the *CaDMR1* in chromosome 5, it could be possible that the same resistance gene was identified. The mechanism of homoserine-induced resistance remains unclear and warrants further gene validation in future studies. Genes coding for serine/threonine protein kinases and leucine rich repeat (LRR) receptors showed higher expression in the current study consistent with previous reports (Siddique et al., 2019; Du et al., 2021; Kang et al., 2022a) confirming their involvement in host defense against infection by *P. capsici*. The observed higher expression of LRR-receptor-like serine/threonine-protein kinase *FLS2* in the roots of chili pepper in this study relates to the previous report on the role of flagellin perception *FLS2* gene in Arabidopsis (Chinchilla et al., 2006). Overall, results in the present study supported the role of HSK and amino acid metabolism in the regulation of plant immunity (Heinemann and Hildebrandt 2021; Moormann et al., 2022; Zeier, 2013) for resistance to pathogen attack by *P. capsici* in chili pepper.

Genes with functions related to systemic acquired resistance (SAR) and defense responses to fungi and other organisms were observed to have high gene expression in the resistant cultivar (CM-334) in multiple tissues across various time vs. time comparisons. As a form of inducible resistance, SAR renders a long-lasting systemic immunity against diverse pathogens and is triggered following a previously localized exposure to a pathogen (Choi and Hwang, 2011). The putative lipid-transfer protein DEFECTIVE INDUCED RESISTANCE 1 (*DIR1*), a member of the lipid transfer proteins (LTPs) and involved in SAR, demonstrated higher expression in the 72h/0h, 24h/0h, and 72h/24h contrasts in all tissue samples for CM-334 in the current study (**Table 3**). Consistent with these observations, the higher expression of *DIR1* has been previously identified in the leaf tissues of two pepper landraces in response to plant infection to *P. capsici* (Rabuma et al., 2022). Recently, the overexpression of a *DIR1* gene in Arabidopsis was observed to enhance response to biotic stress by regulating genes related to defense against diseases and the formation of flavonoids (Duan et al., 2024). LTPs such as *DIR1* may function as either a co-signal or translocator for the release of the mobile signals during SAR in plants (Jung et al., 2003). A pepper-specific SAR gene, *CaSAR8.2*, has been previously identified as a genetic marker for plant infection, and an elicitor of abiotic and environmental stresses in *C. annuum* (Lee and Hwang, 2003). Kaur et al. (2024) previously identified an *SAR8.2*-protein coding gene ~470 kb downstream as a candidate gene for SNP *S5_14665044* detected using multi-locus association mapping approaches in a diverse population of *C. annuum* and *C. annuum* x *C. frutescens* hybrids. In another study, *SAR8.2* was identified in the 27.3–29.2 Mb region of chromosome 5 (within the *Ext-Pc5.1* region identified by Du et al. (2021)) as *CASAR82A*, suggesting its important role as a potential candidate gene for resistance to *P. capsici* (Siddique et al., 2019). No other SAR gene has been identified in chromosome 5 in the current study; however, various genes within the *Ext-Pc5.1* region including a receptor kinase-like protein, *Xa21*, located < 0.90 Mb downstream of the SNP identified by Kaur et al. (2024) and a kunitz trypsin inhibitor 5 that induces programmed cell death in Arabidopsis (Li et al., 2008) were observed to have high gene expression across the R vs. S contrasts for all tissues infected by *P. capsici*. These specific genomic regions with known resistance genes can be targeted further for molecular marker-assisted breeding and selection to improve disease resistance in current pepper germplasm.

Genes with roles related to the formation of cell wall, phenylpropanoid biosynthesis, ethylene activated signaling pathway, and mitogen activated protein kinase (MAPK) showed high gene expression exclusively in the stems in the R vs. S contrast across various times of infection. As the primary barrier of defense, the cell wall provides the pepper plants with a preliminary defense and signal perception against pathogen infection through various detection and defense components (Lei et al., 2023). In Arabidopsis mutations in cellulose synthases altered the disease response to the fungus *Plectosphaerella cucumerina* (Hernandez-Blanco et al., 2007). Cellulose synthase-like D3 (*CSLD3*) and the xyloglucan endotransglucosylase/hydrolase protein 15-like showed in higher expression the stem at 24h/0h in the current study. While the function of *CSLD3* has not been well characterized in *Capsicum* spp., the overexpression of this gene in cotton (*Gossypium hirsutum*) has been observed to restore cell elongation and cell wall integrity by enhancing primary cellulose production (Hu et al., 2019). The pathogen damage to the cell wall triggers phenylpropanoid pathway possibly leading to lignin biosynthesis and thereby producing various plant defense metabolites (Yadav et al., 2020). The involvement of phenylpropanoid biosynthesis pathway has been previously demonstrated in the whole root transcriptome of pepper in response to *P. capsici* (Li et al., 2020) and in the leaf and sheath tissues of rice (*Oryza sativa*) upon insect herbivory (Li et al., 2021). Another class of enzymes, the MAPKs, are involved in signaling plant defense against pathogen attack through receptors/sensors that transduce extracellular stimuli into intracellular responses (Meng and Zhang, 2013). In the resistant host–pathogenic microbe pathosystem, the resistant hosts directly induce pattern-recognition receptor (PRR)-triggered immunity (PTI), such as calcium ion (Ca^2+^) influx, reactive oxygen species (ROS) production, and MAPK activation (Ding et al., 2022). Overall, our results also demonstrate a layer of tissue-specific mechanisms to combat pathogen attack in the *Capsicum-P. capsici* pathosystem.

**Figure 5:**
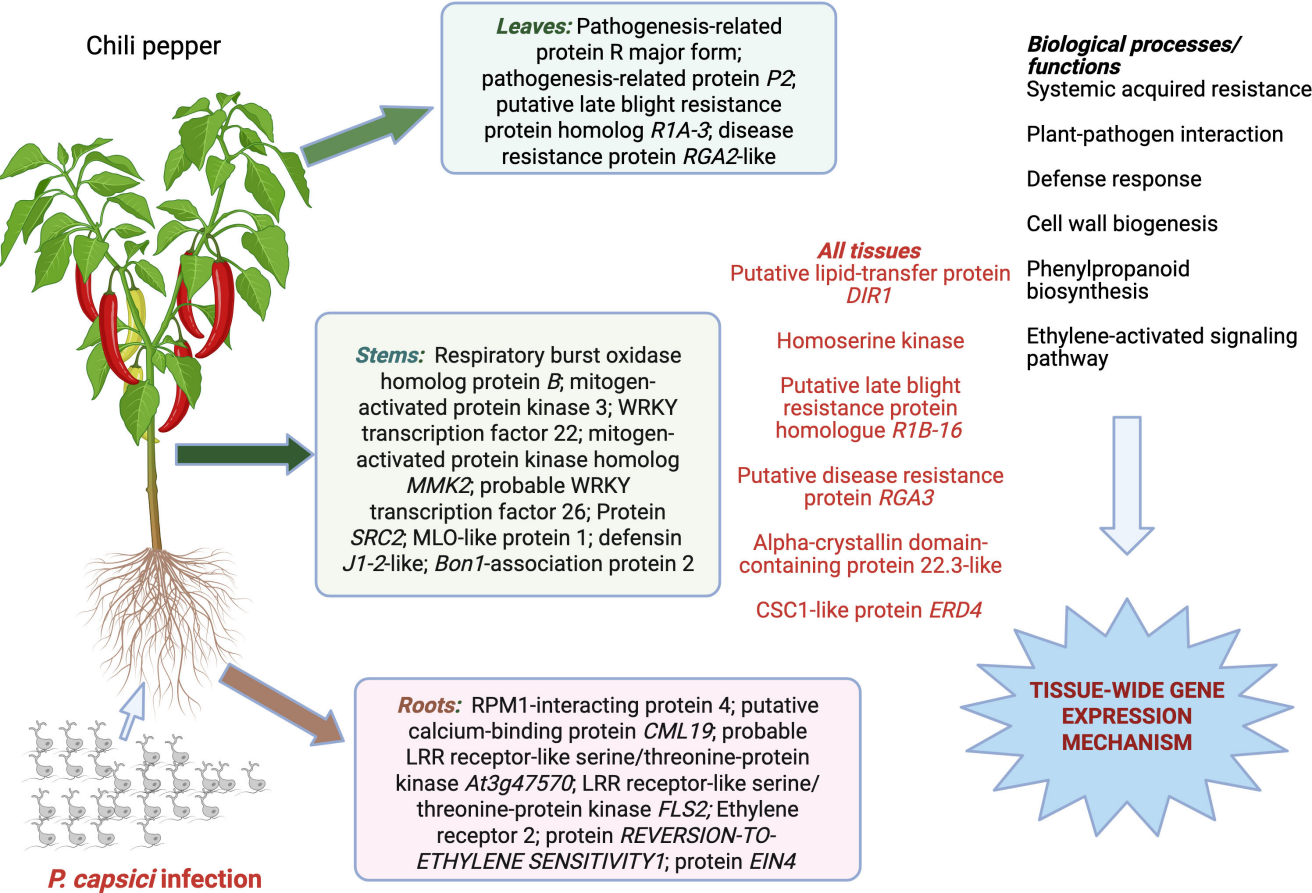
Model for *Phytophthora capsici* resistance in chile pepper derived from comparative transcriptome analyses of roots, stems, and leaf tissue samples. RNA-seq revealed a tissue-wide resistance mechanism conferred by genes that consistently showed higher expression across all tissue samples. Figure created using BioRender (https://www.biorender.com/).

Current results from RNA-seq highlight various genes showing constant higher expression levels across various tissues under oomycete infection in *C. annuum* L. (**Figure 5**). Among these genes, *DIR1*, homoserine kinase, disease resistance proteins *R1B-16* and *RGA3*, and *CSC1*-like proteins consistently showed high expression across all tissues, indicating effector-triggered tissue-wide mechanisms of disease resistance against *P. capsici.* Altogether, resistance to *P. capsici* infection in chili pepper results from the active expression of genes involved in various biological pathways related to the biosynthesis of amino acids, responses to biotic stresses, plant defense signaling, and adaptation. Overall, our results suggest that *P. capsici* resistance could result from the active expression of genes involved in various plant innate defenses such as PTI and effector-triggered immunity. These genes could be leveraged for developing broad-spectrum resistance against *P. capsici* in current *Capsicum* germplasm.

## 5 Conclusions

RNA-seq of resistant and susceptible chili pepper varieties under infection by *P. capsici* and three different tissue types (leaves, stems, and roots) revealed a multitude of genes that showed higher and lower expression. The genes with higher expression have functions related to homoserine kinase activity, biogenesis of the cell wall, biosynthesis of phenylpropanoid, ethylene-activated signaling pathway, mitogen-activated protein kinase, systemic acquired resistance, plant pathogen interaction, and defense response, among others. Transcriptome analyses supported the role of chromosome 5 as a major genomic region harboring resistance genes, particularly homoserine kinase, which showed higher expression across all tissues. The genes with higher expression across all tissue samples indicate the presence of potential tissue-wide gene expression mechanism that exists in chili pepper. One limitation of the current study is the absence of qRT-PCR validation for genes showing differences in expression levels. The robustness of these results, however, could be supported through using stringent statistical criteria (Benjamini-Hochberg False Discovery Rate) in identifying genes with higher or lower expression. Gene expression profiling will serve as a basis for future studies linking gene expression to resistance to *P. capsici*. The existence of tissue-wide genetic mechanisms as revealed by transcriptomic profiling can be used for the breeding and selection of *P. capsici*-resistant chili pepper cultivars.

## Data Availability

The data presented in this study are publicly available. The raw FASTQ files of the transcriptomes can be found here: https://www.ncbi.nlm.nih.gov, accession PRJNA1256908.

## Supplementary material

Supplementary Figure 1 Multidimensional scaling (MDS) or Principal coordinates analysis (PCoA) for **(A)** roots, **(B)** stems, and **(C)** leaves under *Phytophthora capsici* infection demonstrating clustering based on resistance and susceptibility of varieties across various times of infection for the root and stem tissue samples. An apparent overlap of clusters was observed for the leaves.

Supplementary Figure 2 Network of Significantly Enriched GO Functions in Resistant (CM-334) vs Susceptible (Early Jalapeno) samples from different tissues. Results genes with significant high and lower expression are shown. The size of a function node is proportional to the number of differentially expressed (DE) genes annotated with a given function in all the comparisons. The DE genes (small nodes) are shown connected to their corresponding functional categories. Function nodes are connected through shared DE genes. DE gene nodes are colored according to their occurrence in different contrasts. A function node is colored by the presence of DE genes annotated with that function in different contrasts, independently of the enrichment level of the function in each contrast.

Supplementary Table 1 List of software tools and packages used for RNA-sequencing analyses.

Supplementary Table 2 Total number of reads, percentage of pseudoaligned reads, number of unique sequences, and percentage of unique sequences identified using Kallisto.

Supplementary Table 3 Different comparisons of transcriptome profiles performed for *C. annuum* under *P. capsici* infection.

Supplementary Table 4 Top 100 genes with higher expression for *C. annuum* under *P. capsici* infection (Resistant vs Susceptible) across all tissues.

Supplementary Table 5 Top 100 genes with higher expression for *C. annuum* under *P. capsici* infection (tissue vs. tissue) at 0 hpi.

Supplementary Table 6 Top 100 genes with higher expression for *C. annuum* under *P. capsici* infection (tissue vs. tissue) at 24 hpi.

Supplementary Table 7 Top 100 genes with higher expression for *C. annuum* under *P. capsici* infection (tissue vs. tissue) at 72 hpi.

Supplementary Table 8 Top 100 genes with higher expression for *C. annuum* under *P. capsici* infection (tissue vs tissue) in CM-334 (Resistant variety).

Supplementary Table 9 Top 100 genes with higher expression for *C. annuum* under *P. capsici* infection in the roots (time vs. time).

Supplementary Table 10 Top 100 genes with higher expression for *C. annuum* under *P. capsici* infection (time vs time) in the stems.
