# Supplementary material for: Transcriptome profiling reveals tissue-wide gene expression in chili pepper (Capsicum annuum L.) under infection by Phytophthora capsici
Source: Front Plant Sci. 2026 Feb 26;17:1745952. doi: 10.3389/fpls.2026.1745952 (PMC12979562; doi:10.3389/fpls.2026.1745952)
Supplement: Supplementary Figure 1 — Multidimensional scaling (MDS) or Principal coordinates analysis (PCoA) for (A) roots, (B) stems, and (C) leaves under Phytophthora capsici infection demonstrating clustering based on resistance and susceptibility of varieties across various times of infection for the root and stem tissue samples. An apparent overlap of clusters was observed for the leaves. [file Supplementaryfile1.docx]

**B**

**A**

**C**


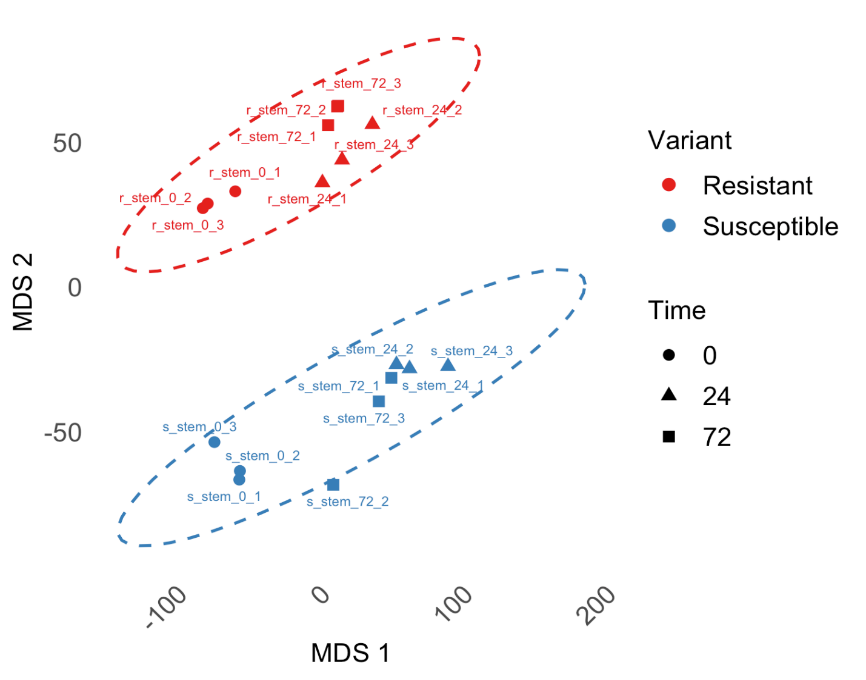

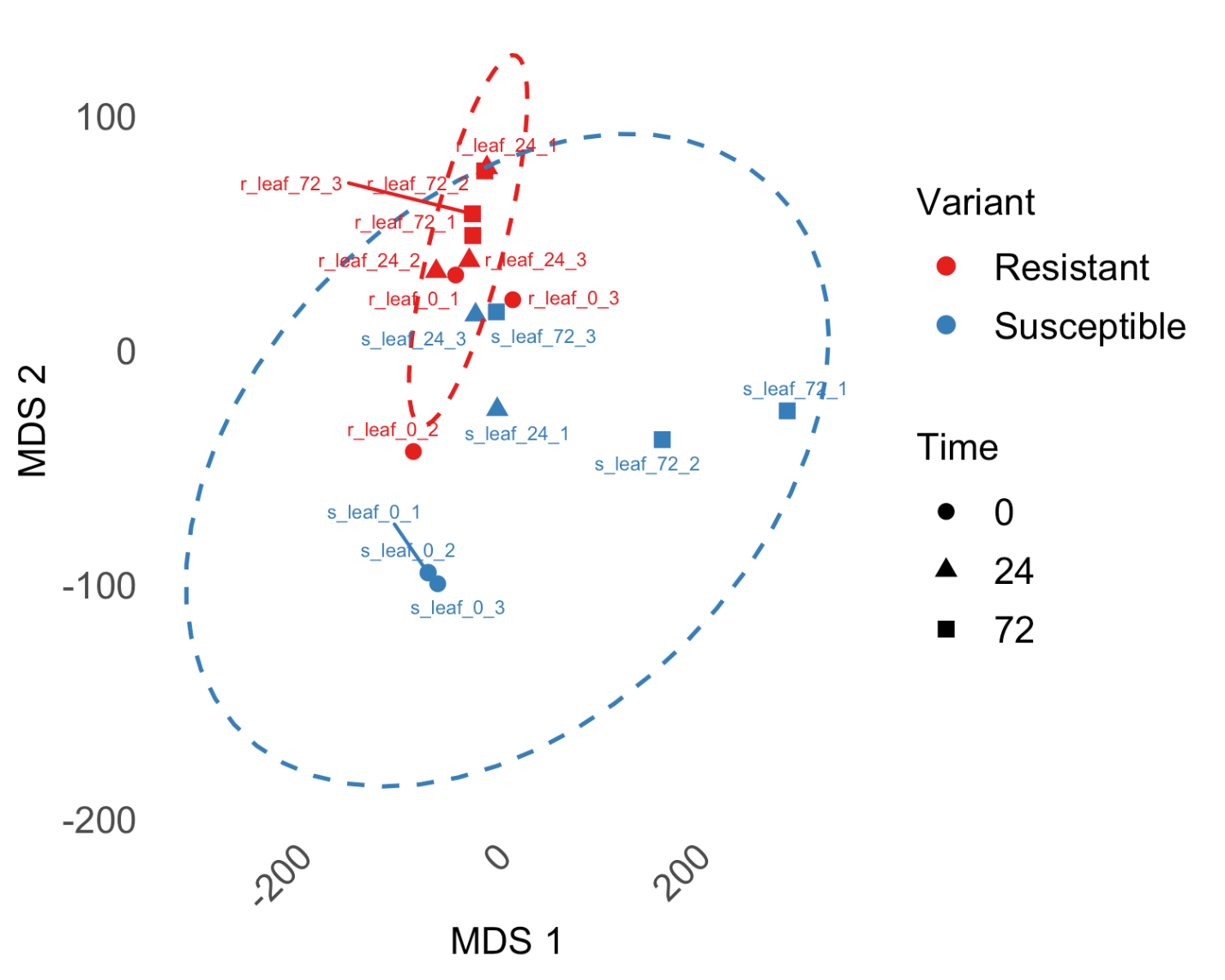

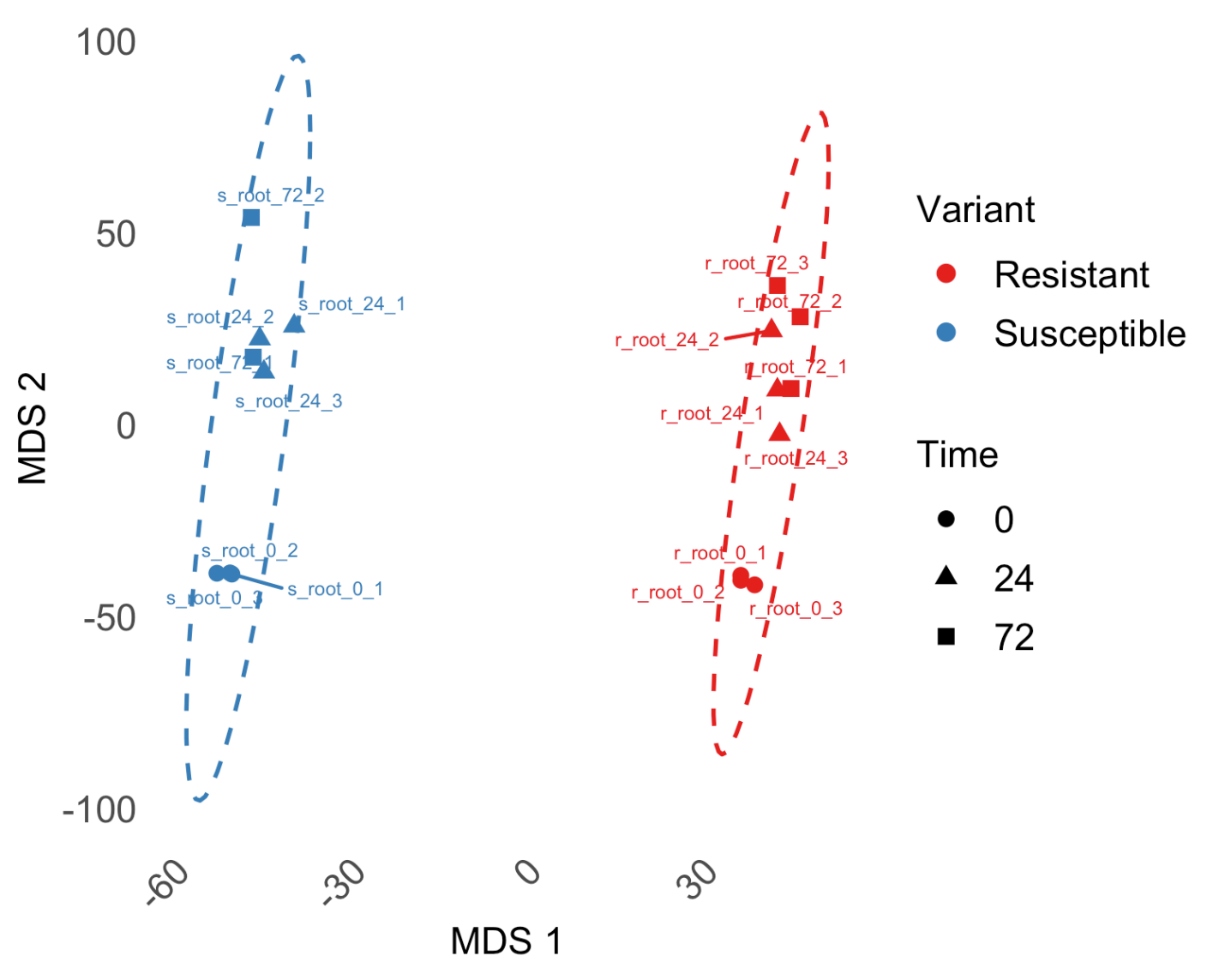


**Supplementary Fig. 1**. Multidimensional scaling (MDS) or Principal coordinates analysis (PCoA) for (A) roots, (B) stems, and (C) leaves under *Phytophthora* *capsici* infection demonstrating clustering based on resistance and susceptibility of genotypes across various times of infection for the root and stem tissue samples. An apparent overlap of clusters was observed for the leaves.


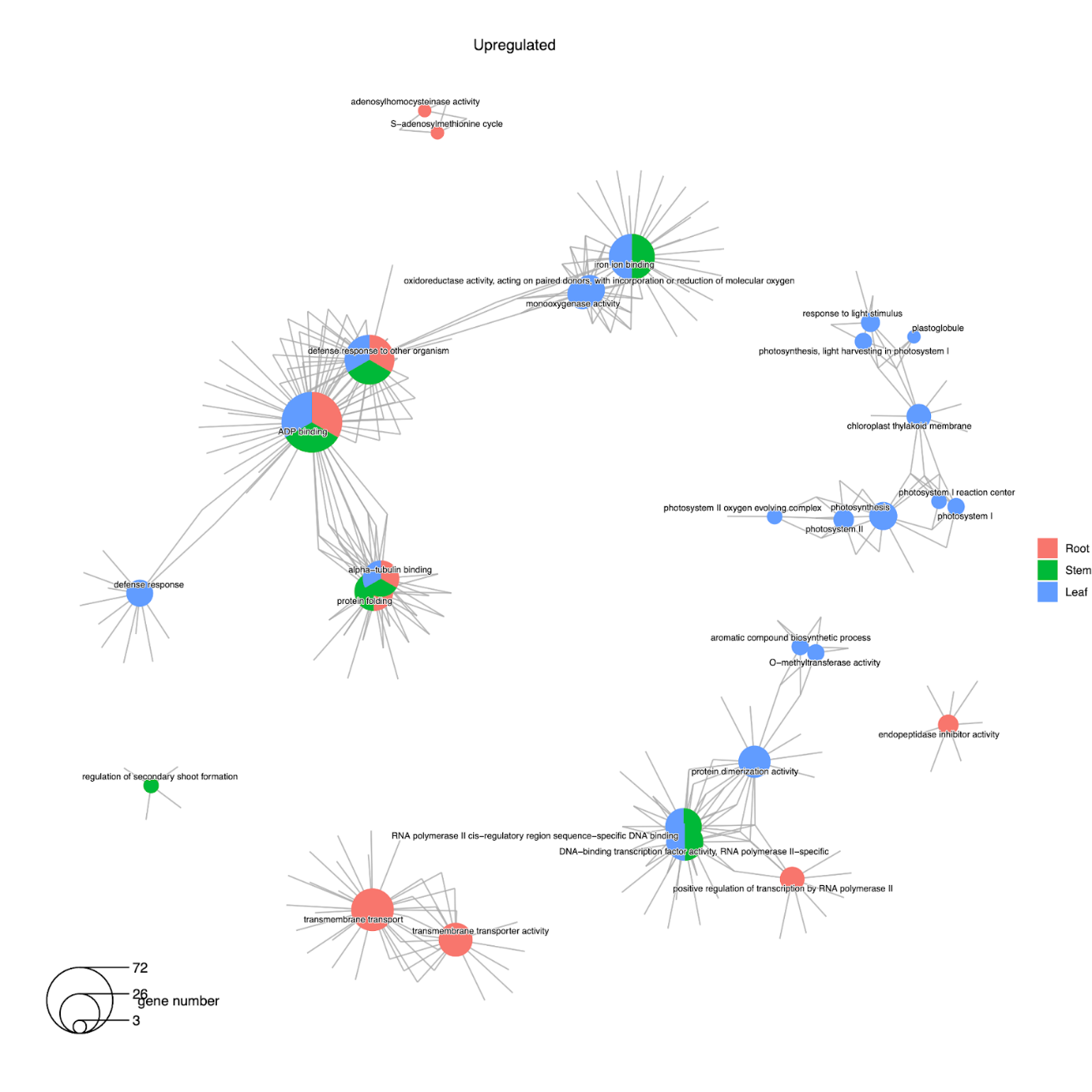


**Supplementary Fig. 2**. Network of Significantly Enriched GO Functions in Resistant (CM-334) vs Susceptible (Early Jalapeno) samples from different tissues. Results from upregulated and downregulated genes are shown. The size of a function node is proportional to the number of differentially expressed (DE) genes annotated with a given function in all the comparisons. The DE genes (small nodes) are shown connected to their corresponding functional categories. Function nodes are connected through shared DE genes. DE gene nodes are colored according to their occurrence in different contrasts. A function node is colored by the presence of DE genes annotated with that function in different contrasts, independently of the enrichment level of the function in each contrast.
